# Unveiled feather microcosm: feather microbiota of passerine birds is closely associated with host species identity and bacteriocin-producing bacteria

**DOI:** 10.1038/s41396-019-0438-4

**Published:** 2019-05-24

**Authors:** Veronika Gvoždíková Javůrková, Jakub Kreisinger, Petr Procházka, Milica Požgayová, Kateřina Ševčíková, Vojtěch Brlík, Peter Adamík, Petr Heneberg, Jiří Porkert

**Affiliations:** 1 Faculty of Agrobiology, Food and Natural Resources, Department of Animal Science, Czech University of Life Sciences, Kamýcká 129, 165 00 Prague-Suchdol, Czech Republic; 2 Institute of Vertebrate Biology, Czech Academy of Sciences, Květná 8, 603 65 Brno, Czech Republic; 3 Faculty of Science, Department of Zoology, Charles University, Viničná 7, 128 44 Prague, Czech Republic; 4 Faculty of Science, Department of Zoology, Palacký University, 17. listopadu 50, 771 46 Olomouc, Czech Republic; 5 Third Faculty of Medicine, Charles University, Ruská 87, 100 00 Prague, Czech Republic; 6 Home address: Gočárova třída 542/12, 500 02 Hradec Králové, Czech Republic

**Keywords:** Microbial ecology, Antimicrobials, Biodiversity

## Abstract

The functional relevance of microbiota is a key aspect for understanding host–microbiota interactions. Mammalian skin harbours a complex consortium of beneficial microorganisms known to provide health and immune-boosting advantages. As yet, however, little is known about functional microbial communities on avian feathers, including their co-evolution with the host and factors determining feather microbiota (FM) diversity. Using 16S rRNA profiling, we investigated how host species identity, phylogeny and geographic origin determine FM in free-living passerine birds. Moreover, we estimated the relative abundance of bacteriocin-producing bacteria (BPB) and keratinolytic feather damaging bacteria (FDB) and evaluated the ability of BPB to affect FM diversity and relative abundance of FDB. Host species identity was associated with feather bacterial communities more strongly than host geographic origin. FM functional properties differed in terms of estimated BPB and FDB relative abundance, with both showing interspecific variation. FM diversity was negatively associated with BPB relative abundance across species, whereas BPB and FDB relative abundance was positively correlated. This study provides the first thorough evaluation of antimicrobial peptides-producing bacterial communities inhabiting the feather integument, including their likely potential to mediate niche-competition and to be associated with functional species-specific feather microbiota in avian hosts.

## Introduction

Intensive microbiome studies across a range of ecosystems and animal taxa have revealed apparent co-evolution between microbes and their hosts [1, 2]. As such, investigations of host–microbiota interactions could prove fundamental to our understanding of host physiology [3], immunity [4], ecology [5, 6] and evolution of life history traits [7]. The past decade has witnessed extensive investigation into the microbiota of mammals and other vertebrates [8]. Recently, however, there has been increasing interest in the microbiota of birds. Despite a dominance of studies focused on commercially exploited species [9, 10], free-living birds and their wild microbiota have become a pivotal theme in avian microbiome research. Most of these studies have focused on gut microbiota, with New World vultures showing a highly conserved and selective gut microbiota resulting from diet specialisation [11], co-diversification of gastrointestinal microbiota and phylogeny in passerines [12] and temporal stability and transgenerational transfer of gut microbiota in a socially living bird [13]. Aside from three recent studies [14–16], however, the microbiota inhabiting skin and its derivatives in free-living birds have remained largely neglected.

Recently, a number of studies have highlighted the importance of vertebrate skin microbiota. Skin microbiota have been shown to affect skin health/disease balance [17], provide antimicrobial protection to progeny [18] and even to regulate expression of innate immune genes in humans and mice [19, 20]. In amphibians, skin microbiota communities have been found to protect anuran and caudate amphibians against chytrid fungus infection and other invading pathogens [21, 22]. Feathers are unique keratin skin derivatives, representing an important evolutionary novelty of theropod dinosaurs and their recent descendants, birds [23]. Previous research using culture-based methods has revealed the presence of diverse microbiota inhabiting the feather surface, and has suggested links between these bacterial communities and heath- or condition-related traits in avian hosts [24–26]. To date, however, only three detailed studies using deep 16S rRNA sequencing have been published on microbiota inhabiting skin and its derivatives in free-living birds [14–16].

In the present study, we investigate feather microbiota (FM) diversity and functional properties in free-living passerine birds, with a special focus on feather damaging bacteria (FDB) and bacteriocin-producing bacteria (BPB). Up to now, the vast majority of culture-based feather microbial assemblage studies have examined FDB synthesising a wide range of keratinases capable of degrading keratinous substrates [27], including feathers [28]. FDB have been shown to impair feather structural quality in vitro [29–31], colour-signalling function [32, 33] and even host immunity [26] in free-living birds. Other studies, however, have documented no detrimental effect of FDB on feather wear quality [34, 35] or a positive synergistic effect, whereby FDB-produced keratinase amplifies the potential of preen gland secretions to eliminate specific FDB from feathers [36]. Moreover, some FDB have been documented as simultaneously producing antibiotics and bacteriocins [37]. Bacteriocins produced by BPB are ribosomally synthesised peptides either antagonising individual bacterial strains [38, 39], or may be non-selective targeting any kind of microorganism [40, 41]. It would appear, therefore, that FDB do not have to be only members of FM impairing host’s feather integument. Alternatively, FDB able to produce bacteriocins can also act as beneficial symbionts for the host maintaining niches with nearly identical dominant strains, while out-competing individual microbial strains, or may be broadly antibiotic for a wide spectrum of microorganisms in FM including pathogens. BPB have been identified in the human and mammalian gastrointestinal tract, where they mediate niche-competition and maintain commensal community structure [42, 43]. In birds, BPB have only been detected in one species to date, the hoopoe (*Upupa epops*), where they were identified on both the surface and in secretions of the single bird sebaceous gland, the preen gland [44–46]. Since BPB may be transmitted from the surface of the preen gland to the feathers via preening [47], we hypothesise that the occurrence of BPB may shape and maintain host-specific FM in birds.

Here, we investigate the FM of seven free-living passerine birds using high throughput 16S rRNA sequencing and test whether feather microbiota diversity and composition is predicted by host species identity and/or its geographic origin. Furthermore, using a bioinformatics approach combining information obtained from public databases (BAGEL4, SILVA) with extensive literature mining, we estimate relative BPB and FDB abundances within FM profiles. This sort of information allowed us to investigate possible correlation between BPB and FDB relative abundances. Assuming that FDB primarily produce keratinases and only limited fraction of FDB can simultaneously produce bacteriocins, we may expect antagonism between BPB and FDB predicting negative correlation between BPB and FDB. Alternatively, as there might be considerable subset of FDB capable to produce bacteriocins, opposite direction of correlation is possible to be expected. Furthermore, based on ability of BPB to produce bacteriocins with broad and also specific antimicrobial potentials, we predict that there will be association between BPB and FM alpha diversity. Specifically, if we consider BPB as broad range non-selective antagonists, FM alpha diversity is expected to decrease with increasing BPB relative abundance. Alternatively, if taking into account BPB producing bacteriocins with antimicrobial activity specific to rather narrow range of bacteria, this may result in complex competition patterns keeping variety of niches not fully occupied and causing increase in FM alpha diversity with increasing BPB relative abundance.

## Material and methods

### Ethical statement

See Supplementary Methods.

### Study species and feather sample collection

Chest contour feathers (approx. 10–15) were collected from two resident and five long-distance migratory passerines (Supplementary Table S1) at different localities within the Czech Republic (Supplementary Fig. S1). To control for the potentially confounding effect of breeding period on FM, individuals of each species were mist-netted at the beginning of their breeding season during February–April 2015. To avoid feather contamination caused by handling, chest contour feathers were plucked directly from each individual trapped in a mist net using microbial DNA-free forceps (i.e., the forceps tip was dipped into ethanol and passed through a flame). The feathers were immediately put into a sterile 1.8 mL cryotube (Deltalab, Barcelona, Spain) and kept cool until delivery to the laboratory, where the samples were subsequently stored at −20 °C until DNA extraction.

### DNA extraction and 16S rRNA gene amplicon sequencing

To assess FM, microbial genomic DNA from whole feather samples was aseptically extracted using the RTP^®^ Bacteria DNA Mini kit (STRATEC Molecular, Berlin, Germany), following Protocol 5 of the isolation kit (Isolation of bacterial DNA from tissue biopsies).

16S rRNA amplicon libraries were prepared and sequenced based on protocols already described in previous studies [12, 48]. See Supplementary Methods for full description of 16S rRNA gene amplicon sequencing and bioinformatic processing methods [49–57].

### Bioinformatic estimation of BPB and FDB

To estimate BPB and FDB relative abundances within species-specific FM, all 16S rRNA sequences for BPB enlisted in the BAGEL4 database [58] (available at: http://bagel4.molgenrug.nl/databases.php), for bacterial species with documented keratinolytic activity listed in the most recent review [27] and in Supplementary Table S2, were extracted from SILVA (version 128; [59]) and used as a reference. Next, our OTU sequences were mapped against this reference database using the UCLUST algorithm [60] with a 97% similarity threshold corresponding approximately to species-level mapping. Finally, the proportion of mapped high-quality reads for each sample was estimated and used as a proxy for BPB and keratinolytic FDB relative abundance. These steps were repeated separately for each of the three BPB classes producing different types of bacteriocins (i.e., Class I, Class II and Class III; see the BAGEL4 database for details) varying in structure and antimicrobial spectrum [61]. The sequence similarity threshold used for OTU sequence mapping did not affect the general conclusions of our analyses. Indeed, we observed high concordance in terms of sample-specific proportions of mapped reads at the 97 and 95% similarity threshold (Pearson correlation: *r* > 0.8 for all BPB classes and for keratinolytic FDB).

### Statistical analyses

Shannon diversity indices and number of OTUs for individual samples were estimated using rarefaction-based normalised OTU tables (i.e., random sub-setting of read counts per sample was 2299 corresponding to minimal sequencing depth). Subsequently, interspecific differences in microbial diversity were analysed using Analysis of Variance (ANOVA).

Four types of dissimilarity indices, i.e., weighted and unweighted UniFrac [62], Bray-Curtis and a binary version of Jaccard dissimilarity, were used to assess differences in FM composition. Jaccard and unweighted UniFrac dissimilarity only account for OTU presence vs. absence and hence are more sensitive to FM changes driven by rare OTUs than Bray-Curtis and weighted UniFrac dissimilarity. In addition, both unweighted and weighted UniFrac dissimilarity take OTU genetic similarity into account, and hence are more sensitive to community divergence driven by phylogenetically distant bacterial taxa.

Among-sample divergence in FM composition was visualised using Non-metric Multidimensional Scaling (NMDS). We then assessed whether there was any correlation between geographic distance between sampling localities (see Supplementary Table S1 and Fig. S1) and FM dissimilarity using the Mantel test. Next, distance-based Redundancy Analyses (db-RDAs), using species identity and geographic distance as response variables, were employed to assess whether there was any interspecific divergence in FM profile composition, while accounting for clinal microbiota variation between sampling localities and vice versa. Their marginal significance (i.e., the effect of species identity controlling for the effect of spatial variation and vice versa) was subsequently tested using anova.cca. The matrix of geographic distance between sampling localities was scaled using Principal Coordinates of Neighbourhood Matrices (PCNM; [63]), the resulting PCNM score matrix being included in the db-RDA models. To prevent model overfitting, scores of the first three PCNM axes only were considered. In order to visualise the effect of geographic distance vs. interspecific variation on FM composition, we constructed a heatmap for the dominant bacterial OTUs detected in FM (i.e., represented by >reads on average in at least one species) using the R package *NMF* [64].

To test for phylosymbiosis, we used the Procrustean Approach to Cophylogeny (R package *PACo*; [65]). Specifically, we searched for a correlation between the matrix of cophenetic phylogenetic distances of host species and matrices for microbiota composition dissimilarities, both being scaled via Principal Coordinate Analysis (PCoA) prior to PACo analyses. Cophenetic distances were calculated based on 1000 Bayesian trees randomly downloaded from http://birdtree.org/ [66]. Significance testing was based on a comparison of Procrustean sums of squares for the original dataset with the distribution of Procrustean sums of squares for random communities constructed by the PACo R function (*n* = 10,000 permutations). To account for the fact that the species sampled were not equally distributed between geographic localities, FM divergence partitions associated with phylogeny, geography and both of these predictors were estimated using the varpart function (R package *vegan*) using db-RDA models that consider both the effect of phylogeny and geography. To build the db-RDAs, dissimilarity indices scaled by PCoA were included as response variables, while sample location identity, PCNM scores for sample location distances and PCoA gradients in phylogenetic cophenetic distances were considered as potential explanatory variables. To prevent model overfitting, a specific set of explanatory variables for each db-RDA was selected using the forward selection procedure (ordistep function in the R package *vegan*). In the results, we report the proportion of variance explained after adjusting for model complexity (i.e., adjusted *R*^2^).

Finally, we used Markov Chain Monte Carlo Generalized Linear Mixed Models (R package *MCMCglmm*; [67]) to assess potential relationships between FM Shannon diversities and estimated relative abundances of BPB or FDB, while taking into account covariance due to shared phylogenetic ancestry. To improve model convergence, BPB and FDB relative abundances were log transformed prior to calculation. To distinguish between processes operating at the inter and intra-specific level, host-specific means of all variables were included as model predictors, along with deviations of each observation from the within-host average [68–71]. We also included the interaction between intra-specific variation and species identity to test for consistency of intra-specific effects among host species sampled. Host species identity and phylogenetic covariance were modelled as random effects.

To account for uncertainty in phylogenetic reconstruction, we conducted separate MCMC simulations for a random sample of 100 Bayesian phylogenetic trees. See Supplementary Methods for full description of MCMC simulations.

## Results

### Taxonomic composition of feather microbiota

**Fig. 1:**
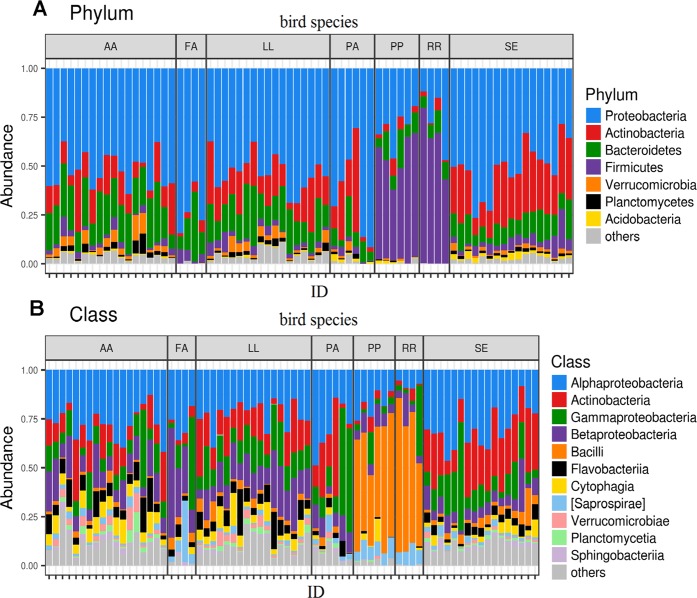
Barplots showing relative abundance of 16S rRNA reads for the (**a**) dominant bacterial phyla and (**b**) classes in particular feather microbiota samples (ID) and host passerine species (AA = *Acrocepalus arundinaceus*, LL = *Locustella luscinioides*, SE = *Sitta europaea*, PA = *Periparus ater*, FA = *Ficedula albicollis*, PP = *Phoenicurus phoenicurus*, RR = *Riparia riparia*)

The FM profiles of almost all passerine species examined, apart from the sand martin (*Riparia riparia*) and common redstart (*Phoenicurus phoenicurus*), were dominated by the phyla *Proteobacteria*, *Actinobacteria* and *Bacteroidetes*, with *Alphaproteobacteria*, *Gammaproteobacteria* and *Betaproteobacteria* dominating at class-level taxonomy (Fig. 1). Interestingly, sand martin and common redstart FMs were dominated by the phylum *Firmicutes* and class *Bacilli* (Fig. 1), with the genera *Streptococcus* and *Lactobacillus* being most prevalent (Supplementary Fig. S2). A detailed summary of the FM taxonomic profiles of each passerine species is presented in Supplementary Fig. S2.

### Feather microbiota richness and diversity

**Fig. 2:**
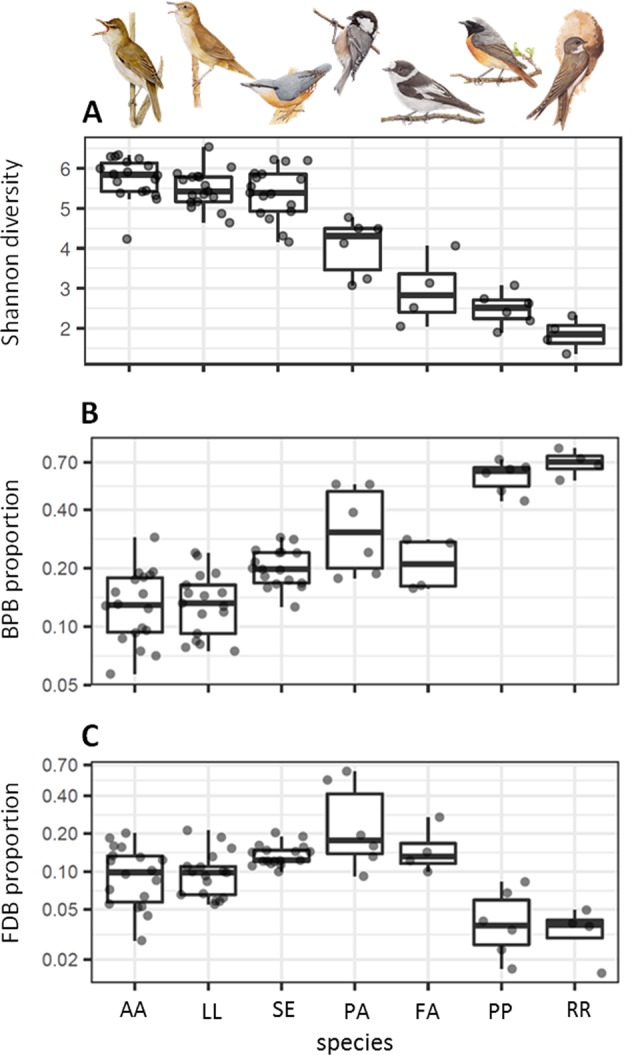
Boxplots of (**a**) Shannon diversities, (**b**) BPB relative abundance and (**c**) keratinolytic FDB relative abundance within the feather microbiota of particular passerine host species (AA = *Acrocepalus arundinaceus*, LL = *Locustella luscinioides*, SE = *Sitta europaea*, PA = *Periparus ater*, FA = *Ficedula albicollis*, PP = *Phoenicurus phoenicurus*, RR = *Riparia riparia*). (Illustrations by Jan Hošek)

FM community alpha diversity varied between individual host species (ANOVA: *F*_6,65_ = 60.51*, p* < 0.0001, *R*^2^ = 0.85; Fig. 2a), with great reed warbler (*Acrocephalus arundinaceus*), Savi’s warbler (*Locustella luscinioides*) and Eurasian nuthatch (*Sitta europaea*) having a more diverse FM than sand martin and common redstart (Fig. 2a).

**Fig. 3:**
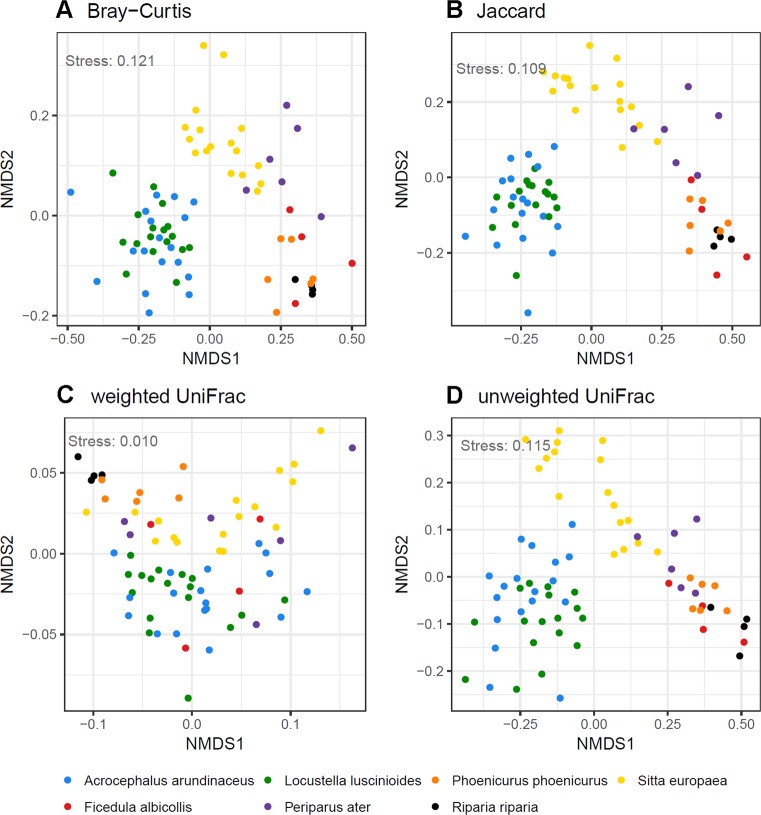
Non-metric Multidimensional Scaling for among-sample divergence in composition (β—diversity) of feather microbial community profiles based on (**a**) Bray-Curtis, (**b**) Jaccard, (**c**) weighted UniFrac and (**d**) unweighted UniFrac dissimilarities. Different colours denote host species identity

NMDS ordination analysis for all dissimilarity indices and PERMANOVA analyses (*p* < 0.0001, *R*^*2*^ range = 0.35–0.42 for all dissimilarity indices) confirmed pronounced interspecific variation in FM composition between host species (Fig. 3a–d). However, we also observed a strong correlation between geographic distance between sampling localities and community-wide dissimilarity in microbial profiles (Mantel tests: *p* < 0.0001, *R*^*2*^ range = 0.38–0.66, for all dissimilarity indices). Consequently, db-RDA models were fitted to assess the extent to which the observed interspecific divergence in FM profiles was confounded by sampling different host species at different sites. The db-RDA analysis revealed significant effect of geography (i.e., sampling site locality) on all types of FM community dissimilarity, except for weighted UniFrac (Table 1). Despite this, interspecific differences in FM composition remained highly significant (*p* < 0.001) when controlling for the effect of geographic distance between sampling localities (Table 1). Three species from our dataset (reed warbler, Savi´s warbler and collared flycatcher) were sampled from a single locality, however, whereas one species (sand martin) was sampled at multiple localities where data for other species were not collected (see Supplementary Table S1 for details). To demonstrate that the sampling scheme and associated db-RDA analyses did not confound the effect of species identity on FM divergence, an additional db-RDA analyses were conducted on a subset of the remaining three species. These analyses consistently supported a stronger effect of host species identity on FM divergence than geographic distance between sampling localities (Supplementary Table S3). Comparable results were obtained when we restricted these analyses to the three localities where we collected data for at least two different species simultaneously sampled at other localities, and when identity of sampling locality instead of geographic distances between sampling localities was treated as a factorial predictor (Supplementary Table S3).

**Table 1:** Db-RDA ANOVA results for feather microbiota divergence associated with host species identity and geographic distance between sampling localities

Dissimilarity index	Variable	df	Variance	*F*	*p*
w. UniFrac	Species	6	0.0003	10.803	0.001
	Between-locality geographic distance	3	0.0000	0.282	0.945
	Residual	62	0.0002		
u. UniFrac	Species	6	0.0230	43.999	0.001
	Between-locality geographic distance	3	0.0010	3.374	0.006
	Residual	62	0.0050		
Bray-Curtis	Species	6	0.0390	75.383	0.001
	Between-locality geographic distance	3	0.0000	2.166	0.042
	Residual	62	0.0050		
Jaccard	Species	6	0.0286	59.264	0.001
	Between-locality geographic distance	3	0.0000	2.938	0.01
	Residual	62	0.0050		

Tests were run on four types of dissimilarity index (weighted and unweighted UniFrac, Jaccard and Bray Curtis)

Variance associated with individual effect, values of *F* statistics and marginal, premeditation-based *p* values are shown

**Fig. 4:**
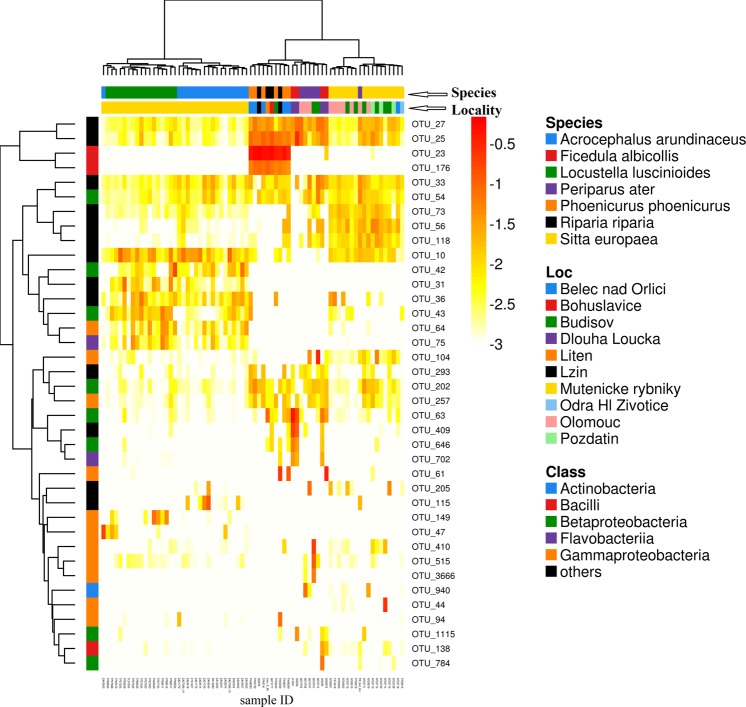
Heatmap denoting abundance of the dominant bacterial OTUs detected in the feather microbiota of individual passerine host species. Both rows and columns are clustered using the Ward algorithm. Identity of host species is indicated by column annotation, whereas row annotations correspond to the taxonomic assignation to the bacterial phyla

In line with the db-RDA analysis, cluster heatmaps of the dominant bacterial OTUs showed host species identity to be a stronger predictor of FM divergence than sampling locality (Fig. 4). In particular, Eurasian nuthatch and coal tit (*Periparus ater*) FM profiles were grouped in single sub-clusters, despite being sampled at different sites, as were common redstarts and sand martins. In addition, hierarchical clustering was able to (almost) perfectly distinguish great reed and Savi’s warblers originating from a single site. The collared flycatcher (*Ficedula albicollis*) was the only species that fell into two distinct sub-clusters (Fig. 4); however, this could not be ascribed to the effect of sample location as the species was only sampled at a single site.

### Effect of host species phylogeny on feather microbiota divergence

**Fig. 5:**
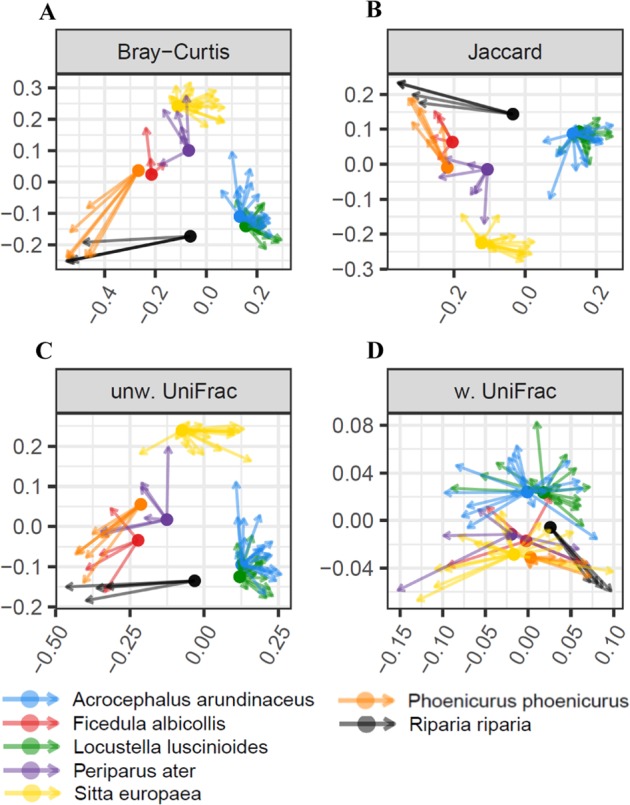
Procrustean superimposition for PCoA‐scaled phylogenetic vs. feather microbiota distance. Feather microbiota divergence was calculated using (**a**) Bray-Curtis, (**b**) Jaccard, (**c**) unweighted UniFrac and (**d**) weighted UniFrac dissimilarities. Different colours denote host species identity

In general, PACo analyses suggested co-divergence between FM composition and host phylogeny (PACo: *p* < 0.0001 for all dissimilarity indices; Fig. 5a–d). Nevertheless, Procrustean superimposition plots suggested a relatively weak correlation for weighted UniFrac (Fig. 5d). Moreover, in line with the NMDS analysis, PACo revealed convergence of two phylogenetically distant species, the common redstart and sand martin (Fig. 5a–c).

According to the db-RDA models, phylogeny explained from 5 to 12% of divergence in FM composition (*p* < 0.01 for all dissimilarity indices) after controlling for FM geographic divergence. Depending on the type of FM dissimilarity indices, geography explained from 2 to 16% of FM divergence (Supplementary Fig. S3). All FM dissimilarity indices (except the unweighted UniFrac), ascribed around 5% of FM divergence to the joint effect of phylogeny and geography.

### Proportion of BPB and keratinolytic FDB in feather microbiota

**Fig. 6:**
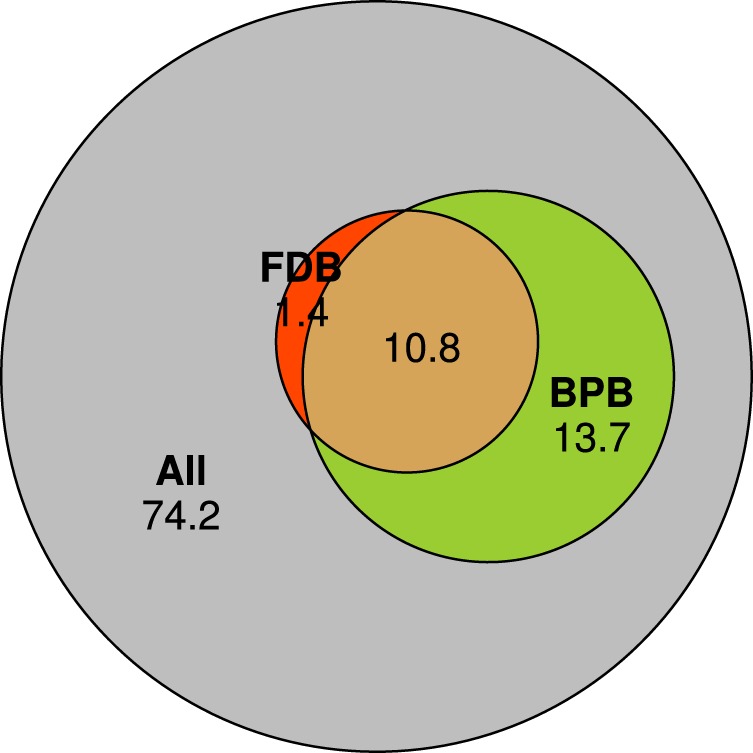
Euler diagram for the relative abundance of bacteriocin-producing (BPB) and keratinolytic (FDB) OTUs within all observed OTUs in the feather microbiota of passerine host species. Numbers in the diagram denote the proportion (%) of unique and shared OTUs

Estimates of the FM fraction capable of producing different bacteriocin classes (i.e., Class I, II or III, see above for details) were highly correlated (Pearson correlation: *r* > 0.8, *p* < 0.0001 for all pair-wise comparisons). The relative abundance of BPB and keratinolytic FDB estimated within all high-quality reads was 24.5% and 12.2%, respectively (Fig. 6). Moreover, 92% (i.e., 10.8% of the overall 12.2%) of all estimated FDB was simultaneously assigned to BPB (Fig. 6). Furthermore, the BPB relative abundance within FM varied dramatically across species (ANOVA: *F*_6,65_ = 29.41, *p* < 0.0001, *R*^2^ = 0.73; Fig. 2b), as did the relative abundance of keratinolytic FDB (ANOVA: *F*_6,65_ = 12.46, *p* < 0.0001, *R*^2^ = 0.53; Fig. 2c). Interestingly, sand martins and common redstarts harboured the highest BPB relative abundance (Fig. 2c), with the class *Bacilli* dominating in both (Fig. 1) and with the prevalence of *Streptococcus* spp. (53 and 45%, respectively) and *Lactobacillus* spp. (9 and 7%, respectively) (Supplementary Fig. S2). Sand martins and common redstarts also had the lowest estimated relative abundance (4%) of keratinolytic FDB compared to other species (Fig. 2c).

**Fig. 7:**
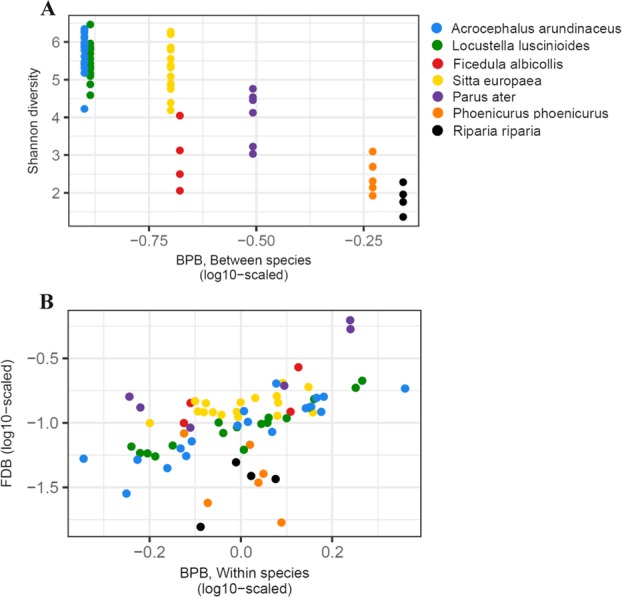
Correlation between (**a**) BPB relative abundance and Shannon diversity and (**b**) relative abundance of BPB and keratinolytic FDB within the feather microbiota of host passerine species. Different colours denote host species identity

MCMCglmms revealed a negative effect of interspecific variation in BPB relative abundance on FM alpha diversity (MCMCglmm: slope = −4.53, 95% CrI = −7.41, −1.54; Fig. 7a). However, this relationship was not supported at the intraspecific level (slope = −0.17, 95% CrI = −1.10, 0.76). DIC was unaffected by presence (DIC = 132.5) or absence (DIC = 132.2) of species-specific effects (i.e., interaction between host species and intraspecific variability in BPB relative abundance) in the model, suggesting that the association between FM alpha diversity and intraspecific variability in BPB relative abundance did not differ between the host species.

In the case of keratinolytic FDB, we found a positive relationship between the relative abundance of FDB and BPB within host species (MCMCglmm: slope = 1.01, 95% CrI = 0.78, 1.24; Fig. 7b); however, this relationship was not supported between host species (MCMCglmm: slope = −0.72, 95% CrI = −1.65, 0.22). A lower DIC for main effect models (DIC = 70.19) compared to models including interactions (DIC = 79.99) suggested a consistent association between BPB within species variation and FDB relative abundance between species.

We observed no association between FM alpha diversity and interspecific (MCMCglmm: slope = 2.62, 95% CrI = −2.12, 7.42) or intraspecific (MCMCglmm: slope = −0.29, 95% CrI = −0.96, 0.39) variation in FDB relative abundance. Main effect models displayed a slightly lower DIC (DIC = 130.0) than those with species-specific interaction (DIC = 131.4), suggesting a lack of any pronounced relationship between host-specific FM diversity and variability in FDB relative abundance.

While H^2^ estimates were low (<0.005 for all models), associated posterior credible intervals were broad (ranging between <0.0001 and 0.8 in all models), implying that data from more species are required for reliable quantification of phylogenetic signal of these traits.

Finally, to explore if the existence of distinct FM phylotype affects associations between FM diversity and BPB and FDB relative abundances, we reran MCMCglmm simulations on a data subset where species whose FM was dominated by the genera *Streptococcus* and *Lactobacillus* (i.e., sand martin and common redstart) were excluded. Consistent with results for the whole dataset, we found a highly supported positive association between relative abundances of FDB and BPB within host species (MCMCglmm: slope = 1.06, 95% CrI = 0.87, 1.23) and no association between FM diversity and FDB relative abundance. Similarly, although significance was not further supported based on posteriori credible intervals in MCMCglmm for the data subset excluding species with specific FM phylotype, the slope of negative correlation between BPB relative abundance and FM alpha diversity at interspecific level remained almost unchanged (MCMCglmm: slope = −4.73, 95% CrI = −17.79, 8.68).

## Discussion

This study provides the first in-depth insights into interspecific diversity and the potential functional significance of FM in free-living passerine bird populations. In doing so, we revealed the underlying role of host species identity in shaping FM diversity and community profiles, with local environment having a weaker effect. Moreover, we observed a high prevalence of estimated BPB within passerine bird FM and pointed out their potential to be associated with species-specific FM diversity. These results suggest that avian feathers, as the most pronounced keratinous skin derivative, harbour species-specific bacterial communities that are likely to produce antimicrobials, thus serving as potentially important host guardians, similar to those on mammalian skin.

These findings are in agreement with the most recent comparative studies confirming that microbiota inhabiting vertebrate skin and its derivatives depend strongly on host-species ecology and environmental factors. Specifically, the interaction of host species identity and environmental conditions at both regional and local scales has been shown to shift diversity and composition of bat skin microbiota [72]. On the other hand, the skin microbiota of 89 tropical frog species sampled across 30 Madagascan sites was mostly determined by host-specific ecomorphology rather than sampling site characteristics [73], with identical outcomes also observed for skin microbial community profiles in temperate frogs [74]. In birds, three captive-bred finch species were shown to harbour highly species-specific skin microbiota, despite experiencing the same environments [75]. Similarly, the skin microbiota of a pelagic seabird, Leach’s storm petrel (*Oceanodroma leucorhoa*), was shown to be independent of microbes from the surrounding environment and mainly affected by sex and individual genetic factors [15]. Finally, a recent study that also used 16S rRNA profiling to compare the FMs of two sympatric lark species revealed horizontal acquisition of feather microbes from the shared environment [14]. Hence, it would appear that while the microbial assemblage of vertebrate integuments is driven to some extent by environmental niche-specific characteristics, host-specific factors have a stronger effect. At this time, however, the actual identity of those factors remains largely unknown in birds and most non-human vertebrates.

The FM of our focal passerine species was dominated by the phyla *Proteobacteria*, *Actinobacteria*, *Bacteroidetes*, *Verrucomicrobia* and *Planctomycetes*, with *Alphaproteobacteria*, *Actinobacteria* and *Gammaproteobacteria* dominant at class-level taxonomy. These results are consistent with a previous study investigating the FM of two sympatric lark species [14]. Our data revealed the existence of an FM phylotype distinct from FM profiles observed in most of our focal species, however, as well as those from previous studies [14, 76]. Interestingly, this phylotype was specific to sand martins and common redstarts, whose feathers were dominated by the phylum *Firmicutes* and the class *Bacilli*, with a high proportion of the genera *Streptococcus* and *Lactobacillus*. The genus *Streptococcus* is a common member of core human skin microbiota and represents a key commensals inhabiting the skin of healthy individuals [17]. Pathological increases in the abundance of streptococci are usually linked to skin damage caused by disease or immunocompromised individuals [77, 78]. In the present study, we can exclude pathological cutaneous infection as a cause of the high prevalence of streptococci in the feathers of these species as we sampled multiple individuals from geographically segregated populations. Furthermore, a recent study [79] analysing skin microbiota across mammals revealed a high abundance (16%) of the genus *Streptococcus* in a chiropteran species, the Indian flying fox (*Pteropus giganteus*). Similarly, the family *Streptococcaceae* accounts for 3 to 24% of the total bacterial community on fish skin [80]. It would appear, therefore, that streptococci are common skin commensals in many free-living vertebrates, having a beneficial rather than pathogenic function for the host [17]. On the other hand, the genus *Lactobacillus* has only been identified as a member of the core skin microbiota of humans [81] and free-living primates [79] to date. Thus, the present study provides first evidence for the occurrence of these potentially beneficial symbiotic microorganisms inhabiting skin derivatives of non-mammalian vertebrates.

Our data suggest a pattern of phylosymbiosis for FMs in phylogenetically related species. Phylosymbiosis postulates that divergence in host-associated microbiota parallels host species phylogeny, which may indicate intensive co-evolution between hosts and their microbiota [82]. However, phylosymbiosis at the community level does not always signify co-evolution. Recent studies have suggested that phylosymbiosis need not be conditioned by co-evolutionary history but by environmental and host-specific factors that may co-vary and contribute significantly to the microbiota assemblage [7, 82, 83]. This may lead to the absence, or an inability to detect, phylosymbiosis patterns in phylogenetically close species [84, 85] or, alternatively, it could result in a convergence of microbiota among unrelated species [86, 87], as observed in this study. We found highly convergent FMs in two phylogenetically distant species, both of which are often in close contact with the soil niche during breeding or foraging. Hence, we argue that, contrary to the strong phylosymbiosis observed for gut microbiota across vertebrate and invertebrate taxa [12, 82], convergence of external integument microbiota in free-living birds (or other vertebrates) is driven by both host-specific factors and, to some extent, by ecological niche sharing, as postulated by the ‘niche-driven microbiota assembly hypothesis’ [14]. Clearly, more studies investigating the factors responsible for shaping and maintaining functional microbiota living on the skin and its derivatives in non-human vertebrates are needed.

Our bioinformatic estimates suggest that all the bird species studied hosted both BPB and FDB in their feathers. In comparison, a previous culture-based study investigating FDB across 154 avian species recorded just 39% of all birds analysed as having FDB in their feathers [25]. We assume that the difference between the two studies may have resulted from the fact that only specific FDB were investigated in the latter study, meaning that the results of the two studies cannot be compared. Moreover, it is worth noting that the use of culture-based techniques commonly leads to underestimation of microbial abundance and false negative results [88]. Of more interest, however, is the discovery of a potentially unique role for BPB within feather microbial communities. We found that relative abundance of estimated BPB was negatively associated with FM alpha diversity in all the passerines studied. This effect was most pronounced in common redstarts and sand martins, whose feather microbial communities were dominated by BPB of the genera *Streptococcus* and *Lactobacillus*, resulting in the lowest FM alpha diversity of all the species studied. As most species within the genera *Streptococcus* and *Lactobacillus* have been shown to produce potent bacteriocins [39, 58, 89, 90], their estimated dominance in the feathers of two focal species suggests a strong potential to shape their feather microbial profiles. On the other hand, great reed and Savi’s warblers had the lowest BPB relative abundances of all focal species, but the highest FM alpha diversities. This is in accordance with previous studies documenting a plethora of BPB within the gut microbiota of vertebrates [91, 92] that are able to significantly augment niche competition [42, 43] and decrease gut microbiota diversity and composition [90, 93]. A few studies have also recorded the presence of resident commensals, including bacteriocin-producing streptococci, as underlying agents maintaining a balanced and healthy human skin microbiota [17]. Moreover, experimental evidence exists for the importance of skin surface symbionts able to switch skin microbiota abundance and diversity in tadpoles [94]. Though evidence exists for the potential of mechanisms based on preening and preen gland secretions to affect FM [16, 36, 95], the present study is the first to point out an association between antimicrobial compound-producing bacterial commensals and FM divergence in free-living birds. However, experimental studies are needed to verify the likely potential of BPB to substantially shape FM. In addition, we documented that after the exclusion of the species that harboured microbial phylotype dominated by the genera *Streptococcus* and *Lactobacillus* from the dataset, regression slope of negative correlation between FM diversity and BPB relative abundance was almost identical, yet its significance was not further supported. Consequently, we cannot unambiguously resolve, whether such decrease of support for the association between BPB relative abundance vs FM alpha diversity was caused by low statistical power or by the effect FM phylotype. More robust comparative datasets are therefore needed to resolve to what extent existence of contrasting FM phylotypes is involved in the negative association between BPB relative abundance and FM alpha diversity.

Recent studies have documented skin microbial commensals protecting the skin against pathogens and detrimental microorganisms in humans [96], amphibians [21, 22, 97] and fish [80]. While there was no negative correlation between BPB and FDB in this study, we observed a positive association between BPB relative abundance and FDB at the intraspecific level. As such, our results differ from those of the most recent research on humans and non-human vertebrate skin microbiota. In the case of potentially harmful FDB, however, it is worth noting that only one *in vivo* study has provided evidence for a negative effect on feather quality [98], while two other studies showed no relationship between FDB and feather wear [34, 35]. These findings partially support our speculation on a positive association between BPB and FDB because the fact that most FDB (mostly from the genera *Bacillus* and *Pseudomonas*) are simultaneously BPB. As such, they are able to produce bacteriocins [99, 100] that may augment niche-competition within FMs and maintain well-balanced feather commensal microbiota. We are aware that our findings are limited to BPB and FDB relative abundance within FM, which precludes an evaluation of any relationship between specific BPB and FDB absolute abundance and *vice versa*. Nevertheless, our findings suggest that the potentially deleterious role of FDB in birds should be re-evaluated and the occurrence of FDB on feathers should not necessarily be interpreted as detrimental, but at least neutral or even beneficial for birds.

For the first time, this study provides support for the hypothesis that feathers, as the most pronounced keratinous skin derivative, harbour species-specific bacterial communities that are able to produce antimicrobials similar to those on mammalian skin. While the positive effect of skin microbiota on host health and immune status has previously only been well documented in humans, the present study significantly extends our knowledge of the functional potential of microbiota inhabiting skin derivatives and outlines avenues for future research on the driving forces behind the evolution of host-integument microbiomes in non-human vertebrates.

## Supplementary information

Supplementary Methods

Table S1

Table S2

Table S3

Table S4

Figure S1

Figure S2

Figure S3

## Data Availability

All sequence data are deposited in the form of fastq files for each sample in the European Nucleotide Archive (ENA). Metadata for these files and their accession numbers are provided in Supplementary Table S4.
