# Supplementary material for: Unveiled feather microcosm: feather microbiota of passerine birds is closely associated with host species identity and bacteriocin-producing bacteria
Source: ISME J. 2019 May 24;13(9):2363–76. doi: 10.1038/s41396-019-0438-4 (PMC6775979; doi:10.1038/s41396-019-0438-4)
Supplement: Supplementary file 1 — Supplementary Methods [file 41396_2019_438_MOESM1_ESM.docx]

**Supplementary Methods**

**Material and Methods**

***Ethical statement***

This study was performed under Animal Welfare License 13060/2014-MZE-17214 of Charles University. Birds were sampled under Permit 47941/ENV/15-2247/630/15, issued by the Ministry of Environment of the Czech Republic.

## **16S rRNA gene amplicon sequencing**

In brief, we amplified V3-V4 16S rRNA using universal Eubacterial primers (S-D-Bact-0341-b-S-17 and S-D-Bact-0785-a-A-21) [49] tagged with 10bp identifiers. After equimolar normalisation and purification of the PCR products, amplicon libraries were prepared using TruSeq Nano DNA Library Preparation kits (Illumina, San Diego, California, USA) and sequenced on a single Illumina MiSeq run using v3 chemistry and 300 bp paired-end reads. Technical PCR duplicates were sequenced for individual DNA samples. As there was high consistency in both FM composition (Procrustean correlation: *r* = 0.97, *p* < 0.0001) and FM diversity (Pearson correlation: *r* = 0.96, *p* < 0.0001) between technical replicates, we merged sequences corresponding to individual samples for downstream analysis.

## **Bioinformatic processing of sequencing data**

Paired-end Illumina reads were merged using PEAR [50]. FASTQ files were demultiplexed and primers trimmed using skewer [51]. Next, we filtered sequences of low quality (max. expected errors per sequence < 1) and de-noised filtered dataset using DADA2 [52]. UCHIME (implemented in the Lotus pipeline; [53]) was used alongside the gold.fna database (available at: http://sourceforge.net/projects/microbiomeutil/files) for detection and elimination of chimeric sequences. The taxonomy of the resulting 16S rRNA variants (hereafter, Operational Taxonomic Units = OTUs) was assigned using RDP classifier and Greengenes reference database (version 13.8., [54]). We considered OTUs assigned as “Chloroplast” (3.2% of read after quality filtering) and those not assigned to any bacterial phylum (0.2% of read after quality filtering) as plant contaminants or PCR/sequencing artefacts, respectively, and excluded them from all downstream analyses. Subsequently, representative sequences were aligned using PyNAST [55] and their maximum-likelihood tree constructed using FastTree [56]. After all filtering steps, the final dataset included 3 700 778 high-quality sequences that were clustered to 17 664 (17 618 non-singleton) OTUs. The number of per-sample high-quality reads ranged between 2 299 and 266 195 (median = 47 781). The resulting OTU tables, sample metadata, OTU trees and taxonomic annotation for individual OTUs were merged into the phyloseq objects [57] for further statistical analysis.

## **Statistical analyses**

***Additional MCMC simulations***

To account for uncertainty in phylogenetic reconstruction, we conducted separate MCMC simulations for a random sample of 100 Bayesian phylogenetic trees (downloaded from <https://birdtree.org/> [66]), representing the species studied. Subsequently, we reported pooled consensual results of these simulations using R package *mulTree* [69]. The simulation for each phylogenetic tree and set of response and explanatory variables was run using two independent MCMC chains, the number of iterations being set at 1 000 000 with burn-in period and thinning interval set at 10 000 and 100 steps, respectively. Chain convergence was acceptable in all cases (Gelman and Rubin m statistic < 1.1 for all simulations). Moreover, effective sample size for merged chains (> 1 950 000 for all estimated parameters) was comparable with the expected sample size given the MCMC chain length (2 000 000; i.e. 100 phylogenetic trees * samples of parameter estimates * 2 chains), indicating negligible effect of autocorrelation on parameter estimates. The strength of phylogenetic signal was quantified using H^2^ statistics, which is comparable to Pagel's lambda [70] and represents the proportion of residual variation and variation associated with all random effect explained by phylogenetic covariance [71]. We used inverse-Gamma distribution with shape and scale parameters equal to 0.01 as prior for random effects and residual variance. Default normal priors were used for fixed effects.
