# Supplementary material for: Unveiled feather microcosm: feather microbiota of passerine birds is closely associated with host species identity and bacteriocin-producing bacteria
Source: ISME J. 2019 May 24;13(9):2363–76. doi: 10.1038/s41396-019-0438-4 (PMC6775979; doi:10.1038/s41396-019-0438-4)
Supplement: Supplementary file 2 — Table S1 [file 41396_2019_438_MOESM2_ESM.docx]

**Table S1.** List of passerine bird species used in this study including their migratory strategies, sampling localities, number of sampled individuals per locality and total number of individuals per species

| **Species** | **Migratory strategy** | **Sampling site locality (GPS coordinates)** | **No of individuals per locality** | **Total No of individuals per species** |
| --- | --- | --- | --- | --- |
| *Sitta europaea* | Resident | Pozdatin (49.24N 16.03E)  Budisov (49.28N 16.00E)  Belec nad Orlici (50.20N 15.94E)  Olomouc (49.63N 17.34E)  Odra Hl Zivotice (49.67N 17.98E) | 3  6  1  6  1 | 17 |
| *Periparus ater* | Resident | Budisov (49.28N 16.00E)  Olomouc (49.63N 17.34E) | 2  4 | 6 |
| *Phoenicurus phoenicurus* | Long-distance migrant | Belec nad Orlici (50.20N 15.94E)  Budisov (49.28N 16.00E) | 5  1 | 6 |
| *Ficedula albicollis* | Long-distance migrant | Dlouha Loucka (49.83N 17.19) | 4 | 4 |
| *Acrocephalus arundinaceus* | Long-distance migrant | Mutenické rybníky (48.54N 17.32E) | 18 | 18 |
| *Locustella lucsinioides* | Long-distance migrant | Mutenické rybníky (48.54N 17.32E) | 17 | 17 |
| *Riparia riparia* | Long-distance migrant | Liten (49.90N 14.15E)  Lzin (49.13N 14.46E)  Bohuslavice (49.94N 18.11E) | 1  2  1 | 4 |
