# Supplementary material for: Unveiled feather microcosm: feather microbiota of passerine birds is closely associated with host species identity and bacteriocin-producing bacteria
Source: ISME J. 2019 May 24;13(9):2363–76. doi: 10.1038/s41396-019-0438-4 (PMC6775979; doi:10.1038/s41396-019-0438-4)
Supplement: Supplementary file 3 — Table S2 [file 41396_2019_438_MOESM3_ESM.docx]

**Table S2.** List of keratinolytic feather damaging bacteria (FDB) whose 16S rRNA sequences were extracted from the SILVA database and subsequently used as reference sequences for the assessment of FDB proportion within species-specific feather microbiotas

| **Bacterial species with documented keratinolytic activity** |
| --- |
| *Bacillus licheniformis* |
| *Bacillus subtilis* |
| *Bacillus pumilus* |
| *Bacillus cereus* |
| *Bacillus circulans* |
| *Bacillus megaterium* |
| *Bacillus thuringiensis* |
| *Bacillus firmus* |
| *Streptomyces pactum* |
| *Streptomyces thermoviolaceus* |
| *Streptomyces albidoflavus* |
| *Streptomyces flavus* |
| *Kocuria rosea* |
| *Kocuria rhizophila* |
| *Staphylococcus hominis* |
| *Staphylococcus epidermidis* |
| *Staphylococcus hemolyticus* |
| *Arthrobacter ilicis* |
| *Micrococcus nishinomyaensis* |
| *Enterococcus faecalis* |
| *Terrabacter terrae* |
| *Fervidobacterium pennavorans* |
| *Pseudomonas fulva* |
| *Pseudomonas stutzeri* |
| *Pseudomonas fluorescens* |
| *Pseudomonas poae* |
| *Pseudomonas stutzeri* |
| *Stenotrophomonas maltophilia* |
| *Janthinobacterium lividum* |
| *Alcaligenes faecalis* |

References denoting sources and produced keratinases responsible for

keratinolytic activity of particular bacterial species can be found in review

of Sharma and Devi 2018 [27].
