# Supplementary material for: Unveiled feather microcosm: feather microbiota of passerine birds is closely associated with host species identity and bacteriocin-producing bacteria
Source: ISME J. 2019 May 24;13(9):2363–76. doi: 10.1038/s41396-019-0438-4 (PMC6775979; doi:10.1038/s41396-019-0438-4)
Supplement: Supplementary file 4 — Table S3 [file 41396_2019_438_MOESM4_ESM.docx]

**Table S3**. Db-RDAs testing the effect of host phylogeny and geography on FM divergence. Models were fitted on only a subset of passerine species that were sampled at multiple localities. We considered either **A)** geographic distances between sampling localities or **B)** locality identity to account for geographic variation. For more details see Statistical analysis section of the main text. Four dissimilarity indices (Bray–Curtis, Jaccard, unweighted and weighted UniFrac) were used for the calculations

|  | | |  | |  | |  | |  | |  |  |
| --- | --- | --- | --- | --- | --- | --- | --- | --- | --- | --- | --- | --- |
| **A)**  **Dissimilarity index** | | | **Variable** | | **df** | | **Variance** | | ***F*** | | ***p*** |  |
| w. UniFrac | Species | | | | 2 | | 0.0021 | | 5.058 | | 0.009 |  |
|  | Geography | | | | 2 | | 0.0008 | | 1.986 | | 0.134 |  |
|  | Residual | | | | 24 | | 0.005 | |  | |  |  |
| u. UniFrac | Species | | | | 2 | | 0.0361 | | 18.637 | | 0.001 |  |
|  | Geography | | | | 2 | | 0.0083 | | 4.288 | | 0.004 |  |
|  | Residual | | | | 24 | | 0.0232 | |  | |  |  |
| Bray-Curtis | Species | | | | 2 | | 0.0484 | | 28.465 | | 0.001 |  |
|  | Geography | | | | 2 | | 0.009 | | 5.271 | | 0.003 |  |
|  | Residual | | | | 24 | | 0.0204 | |  | |  |  |
| Jaccard | Species | | | | 2 | | 0.0377 | | 20.989 | | 0.001 |  |
|  | Geography | | | | 2 | | 0.0101 | | 5.611 | | 0.001 |  |
|  | Residual | | | | 24 | | 0.0216 | |  | |  |  |
|  | | | |  | |  | |  | |  | | |
| **B)** | | | |  | |  | |  | |  | | |
| **Dissimilarity index** | | **Variable** | | **df** | | **Variance** | | ***F*** | | ***p*** | | |
|  | |  | |  | |  | |  | |  | | |
| w. UniFrac | | Species | | 2 | | 0.0018 | | 3.442 | | 0.028 | | |
|  | | Geography | | 2 | | 0.0004 | | 0.762 | | 0.523 | | |
|  | | Residual | | 20 | | 0.0052 | |  | |  | | |
| u. UniFrac | | Species | | 2 | | 0.0412 | | 16.16 | | 0.001 | | |
|  | | Geography | | 2 | | 0.0065 | | 2.551 | | 0.057 | | |
|  | | Residual | | 20 | | 0.0255 | |  | |  | | |
| Bray-Curtis | | Species | | 2 | | 0.0565 | | 28.897 | | 0.001 | | |
|  | | Geography | | 2 | | 0.0087 | | 4.462 | | 0.005 | | |
|  | | Residual | | 20 | | 0.0196 | |  | |  | | |
| Jaccard | | Species | | 2 | | 0.0426 | | 19.171 | | 0.001 | | |
|  | | Geography | | 2 | | 0.0114 | | 5.115 | | 0.002 | | |
|  | | Residual | | 20 | | 0.0222 | |  | |  | | |
