# Supplementary material for: Unveiled feather microcosm: feather microbiota of passerine birds is closely associated with host species identity and bacteriocin-producing bacteria
Source: ISME J. 2019 May 24;13(9):2363–76. doi: 10.1038/s41396-019-0438-4 (PMC6775979; doi:10.1038/s41396-019-0438-4)
Supplement: Supplementary file 5 — Table S4 [file 41396_2019_438_MOESM5_ESM.docx]

**Table S4.** List of all samples used in this study with metadata and associated ENA accession numbers under which particular sequence data in fastq file format are available

| **ID** | **Species** | **Latitude** | **Longitude** | **Locality** | **ENA_accession** | **ENA_experiment** |
| --- | --- | --- | --- | --- | --- | --- |
| DK13980 | Acrocephalus_arundinaceus | 48.54 | 17.32 | Mutenicke rybniky | ERR3093980 | ERX3154807 |
| Z665324 | Acrocephalus_arundinaceus | 48.54 | 17.32 | Mutenicke rybniky | ERR3094035 | ERX3154862 |
| Z791027 | Acrocephalus_arundinaceus | 48.54 | 17.32 | Mutenicke rybniky | ERR3094036 | ERX3154863 |
| ZA10292 | Acrocephalus_arundinaceus | 48.54 | 17.32 | Mutenicke rybniky | ERR3094037 | ERX3154864 |
| ZA21730 | Acrocephalus_arundinaceus | 48.54 | 17.32 | Mutenicke rybniky | ERR3094038 | ERX3154865 |
| ZA21775 | Acrocephalus_arundinaceus | 48.54 | 17.32 | Mutenicke rybniky | ERR3094039 | ERX3154866 |
| ZA21779 | Acrocephalus_arundinaceus | 48.54 | 17.32 | Mutenicke rybniky | ERR3094040 | ERX3154867 |
| ZA24402 | Acrocephalus_arundinaceus | 48.54 | 17.32 | Mutenicke rybniky | ERR3094041 | ERX3154868 |
| ZA24418 | Acrocephalus_arundinaceus | 48.54 | 17.32 | Mutenicke rybniky | ERR3094042 | ERX3154869 |
| ZA24514 | Acrocephalus_arundinaceus | 48.54 | 17.32 | Mutenicke rybniky | ERR3094043 | ERX3154870 |
| ZA27389_14 | Acrocephalus_arundinaceus | 48.54 | 17.32 | Mutenicke rybniky | ERR3094044 | ERX3154871 |
| ZA27389_15 | Acrocephalus_arundinaceus | 48.54 | 17.32 | Mutenicke rybniky | ERR3094045 | ERX3154872 |
| ZA32301 | Acrocephalus_arundinaceus | 48.54 | 17.32 | Mutenicke rybniky | ERR3094046 | ERX3154873 |
| ZA35974 | Acrocephalus_arundinaceus | 48.54 | 17.32 | Mutenicke rybniky | ERR3094047 | ERX3154874 |
| ZA43505 | Acrocephalus_arundinaceus | 48.54 | 17.32 | Mutenicke rybniky | ERR3094048 | ERX3154875 |
| ZA43507 | Acrocephalus_arundinaceus | 48.54 | 17.32 | Mutenicke rybniky | ERR3094049 | ERX3154876 |
| ZA43523 | Acrocephalus_arundinaceus | 48.54 | 17.32 | Mutenicke rybniky | ERR3094050 | ERX3154877 |
| ZA43540 | Acrocephalus_arundinaceus | 48.54 | 17.32 | Mutenicke rybniky | ERR3094051 | ERX3154878 |
| J41821 | Ficedula_albicollis | 49.84 | 17.20 | Dlouha Loucka | ERR3093981 | ERX3154808 |
| J56295 | Ficedula_albicollis | 49.84 | 17.20 | Dlouha Loucka | ERR3093982 | ERX3154809 |
| J56296 | Ficedula_albicollis | 49.84 | 17.20 | Dlouha Loucka | ERR3093983 | ERX3154810 |
| S508576 | Ficedula_albicollis | 49.84 | 17.20 | Dlouha Loucka | ERR3094001 | ERX3154828 |
| TL97355 | Locustella_luscinioides | 48.54 | 17.32 | Mutenicke rybniky | ERR3094009 | ERX3154836 |
| TN26026 | Locustella_luscinioides | 48.54 | 17.32 | Mutenicke rybniky | ERR3094010 | ERX3154837 |
| TP27205 | Locustella_luscinioides | 48.54 | 17.32 | Mutenicke rybniky | ERR3094014 | ERX3154841 |
| TP27300 | Locustella_luscinioides | 48.54 | 17.32 | Mutenicke rybniky | ERR3094015 | ERX3154842 |
| TP27303 | Locustella_luscinioides | 48.54 | 17.32 | Mutenicke rybniky | ERR3094016 | ERX3154843 |
| TP27304 | Locustella_luscinioides | 48.54 | 17.32 | Mutenicke rybniky | ERR3094017 | ERX3154844 |
| TP27305 | Locustella_luscinioides | 48.54 | 17.32 | Mutenicke rybniky | ERR3094018 | ERX3154845 |
| TP27688 | Locustella_luscinioides | 48.54 | 17.32 | Mutenicke rybniky | ERR3094019 | ERX3154846 |
| TP27705 | Locustella_luscinioides | 48.54 | 17.32 | Mutenicke rybniky | ERR3094020 | ERX3154847 |
| TP42472 | Locustella_luscinioides | 48.54 | 17.32 | Mutenicke rybniky | ERR3094024 | ERX3154851 |
| TP63839 | Locustella_luscinioides | 48.54 | 17.32 | Mutenicke rybniky | ERR3094027 | ERX3154854 |
| TP88511 | Locustella_luscinioides | 48.54 | 17.32 | Mutenicke rybniky | ERR3094028 | ERX3154855 |
| TP88515 | Locustella_luscinioides | 48.54 | 17.32 | Mutenicke rybniky | ERR3094029 | ERX3154856 |
| TP88517 | Locustella_luscinioides | 48.54 | 17.32 | Mutenicke rybniky | ERR3094030 | ERX3154857 |
| TP88524 | Locustella_luscinioides | 48.54 | 17.32 | Mutenicke rybniky | ERR3094031 | ERX3154858 |
| TP88529 | Locustella_luscinioides | 48.54 | 17.32 | Mutenicke rybniky | ERR3094032 | ERX3154859 |
| TP88550 | Locustella_luscinioides | 48.54 | 17.32 | Mutenicke rybniky | ERR3094033 | ERX3154860 |
| PA_bez_kro | Periparus_ater | 49.64 | 17.36 | Olomouc | ERR3094000 | ERX3154827 |
| S673747 | Periparus_ater | 49.64 | 17.36 | Olomouc | ERR3094002 | ERX3154829 |
| S673755 | Periparus_ater | 49.63 | 17.40 | Olomouc | ERR3094003 | ERX3154830 |
| S673758 | Periparus_ater | 49.63 | 17.40 | Olomouc | ERR3094004 | ERX3154831 |
| S678701 | Periparus_ater | 49.28 | 16.01 | Budisov | ERR3094006 | ERX3154833 |
| S678710 | Periparus_ater | 49.26 | 15.99 | Budisov | ERR3094007 | ERX3154834 |
| TN48251 | Phoenicurus_phoenicurus | 50.20 | 15.94 | Belec nad Orlici | ERR3094011 | ERX3154838 |
| TN48283 | Phoenicurus_phoenicurus | 50.20 | 15.94 | Belec nad Orlici | ERR3094012 | ERX3154839 |
| TN48299 | Phoenicurus_phoenicurus | 50.20 | 15.94 | Belec nad Orlici | ERR3094013 | ERX3154840 |
| TP29943 | Phoenicurus_phoenicurus | 49.28 | 16.01 | Budisov | ERR3094023 | ERX3154850 |
| TP63739 | Phoenicurus_phoenicurus | 50.20 | 15.94 | Belec nad Orlici | ERR3094025 | ERX3154852 |
| TP63741 | Phoenicurus_phoenicurus | 50.20 | 15.94 | Belec nad Orlici | ERR3094026 | ERX3154853 |
| J82236 | Riparia_riparia | 49.13 | 14.46 | Lzin | ERR3093984 | ERX3154811 |
| Liten_6_86 | Riparia_riparia | 49.90 | 14.15 | Liten | ERR3093985 | ERX3154812 |
| S673777 | Riparia_riparia | 49.94 | 18.11 | Bohuslavice | ERR3094005 | ERX3154832 |
| TK92369 | Riparia_riparia | 49.13 | 14.46 | Lzin | ERR3094008 | ERX3154835 |
| N723708 | Sitta_europea | 49.24 | 16.03 | Pozdatin | ERR3093986 | ERX3154813 |
| N723709 | Sitta_europea | 49.24 | 16.03 | Pozdatin | ERR3093987 | ERX3154814 |
| N723710 | Sitta_europea | 49.24 | 16.03 | Pozdatin | ERR3093988 | ERX3154815 |
| N723711 | Sitta_europea | 49.28 | 16.00 | Budisov | ERR3093989 | ERX3154816 |
| N723712 | Sitta_europea | 49.26 | 15.99 | Budisov | ERR3093990 | ERX3154817 |
| N723714 | Sitta_europea | 49.24 | 16.02 | Budisov | ERR3093991 | ERX3154818 |
| N723715 | Sitta_europea | 49.27 | 15.97 | Budisov | ERR3093992 | ERX3154819 |
| N723716 | Sitta_europea | 49.27 | 15.97 | Budisov | ERR3093993 | ERX3154820 |
| N723718 | Sitta_europea | 50.20 | 15.94 | Belec nad Orlici | ERR3093994 | ERX3154821 |
| N726819 | Sitta_europea | 49.63 | 17.34 | Olomouc | ERR3093995 | ERX3154822 |
| N726915 | Sitta_europea | 49.59 | 17.26 | Olomouc | ERR3093996 | ERX3154823 |
| N726920 | Sitta_europea | 49.59 | 17.25 | Olomouc | ERR3093997 | ERX3154824 |
| N726921 | Sitta_europea | 49.59 | 17.25 | Olomouc | ERR3093998 | ERX3154825 |
| N726922 | Sitta_europea | 49.59 | 17.25 | Olomouc | ERR3093999 | ERX3154826 |
| TP29915 | Sitta_europea | 49.67 | 17.98 | Odra Hl Zivotice | ERR3094021 | ERX3154848 |
| TP29919 | Sitta_europea | 49.28 | 16.01 | Budisov | ERR3094022 | ERX3154849 |
| TX74818 | Sitta_europea | 49.59 | 17.24 | Olomouc | ERR3094034 | ERX3154861 |
