# Supplementary material for: Unveiled feather microcosm: feather microbiota of passerine birds is closely associated with host species identity and bacteriocin-producing bacteria
Source: ISME J. 2019 May 24;13(9):2363–76. doi: 10.1038/s41396-019-0438-4 (PMC6775979; doi:10.1038/s41396-019-0438-4)

Figure S1. Map illustrating the locality of sampling sites. Different letters and colours denote particular sampling sites.

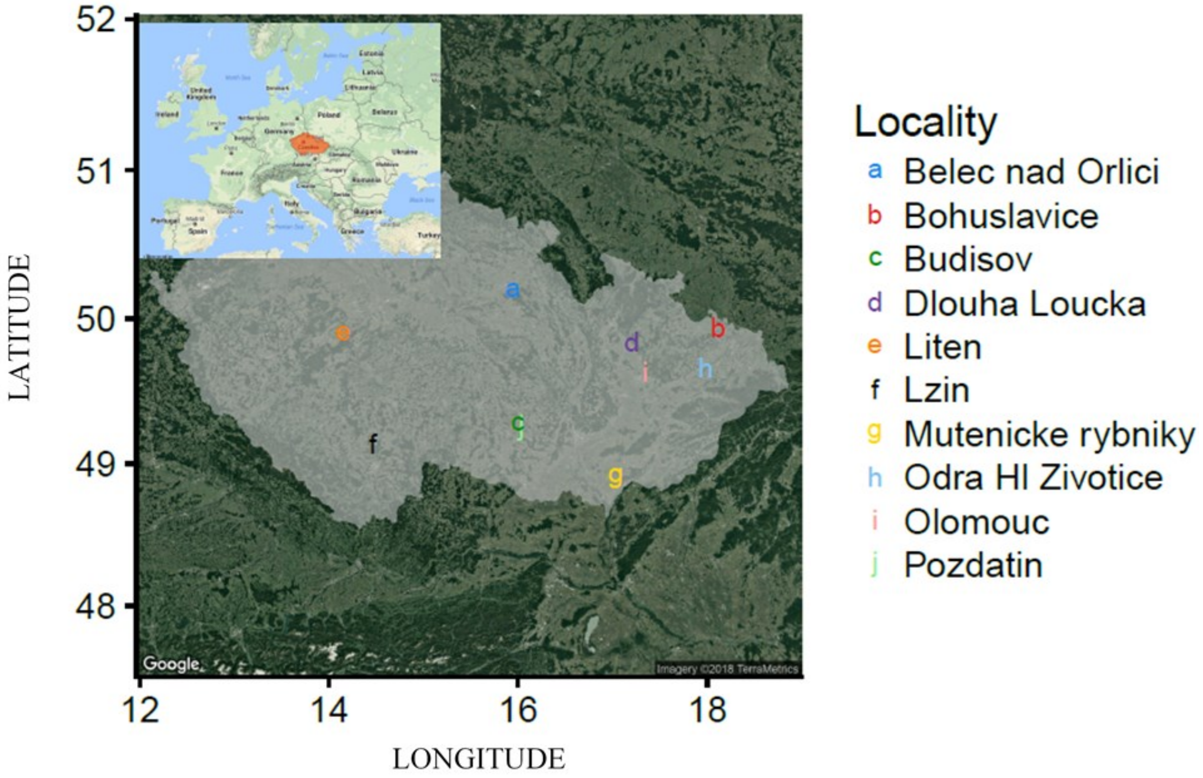

Supplement: Supplementary file 6 — Figure S1 [file 41396_2019_438_MOESM6_ESM.pdf]
