# Supplementary material for: Unveiled feather microcosm: feather microbiota of passerine birds is closely associated with host species identity and bacteriocin-producing bacteria
Source: ISME J. 2019 May 24;13(9):2363–76. doi: 10.1038/s41396-019-0438-4 (PMC6775979; doi:10.1038/s41396-019-0438-4)
Supplement: Supplementary file 7 — Figure S2 [file 41396_2019_438_MOESM7_ESM.pdf]

Figure S2. Set of hierarchical pie charts showing the taxonomic profiles (i.e. average proportions of dominant bacterial taxa) in the feather microbiome of individual passerine species. Pie charts for individual passerine species are available at Figure S2
