# Supplementary material for: Unveiled feather microcosm: feather microbiota of passerine birds is closely associated with host species identity and bacteriocin-producing bacteria
Source: ISME J. 2019 May 24;13(9):2363–76. doi: 10.1038/s41396-019-0438-4 (PMC6775979; doi:10.1038/s41396-019-0438-4)

Figure S3. Adjusted proportion of divergence in FM of passerine birds explained by the effect of phylogeny, geography and their correlation. Calculations are based on db-RDA and varpart function in R package *vegan*. Four dissimilarity indices - Bray–Curtis, Jaccard, unweighted and weighted UniFrac were used for the calculations

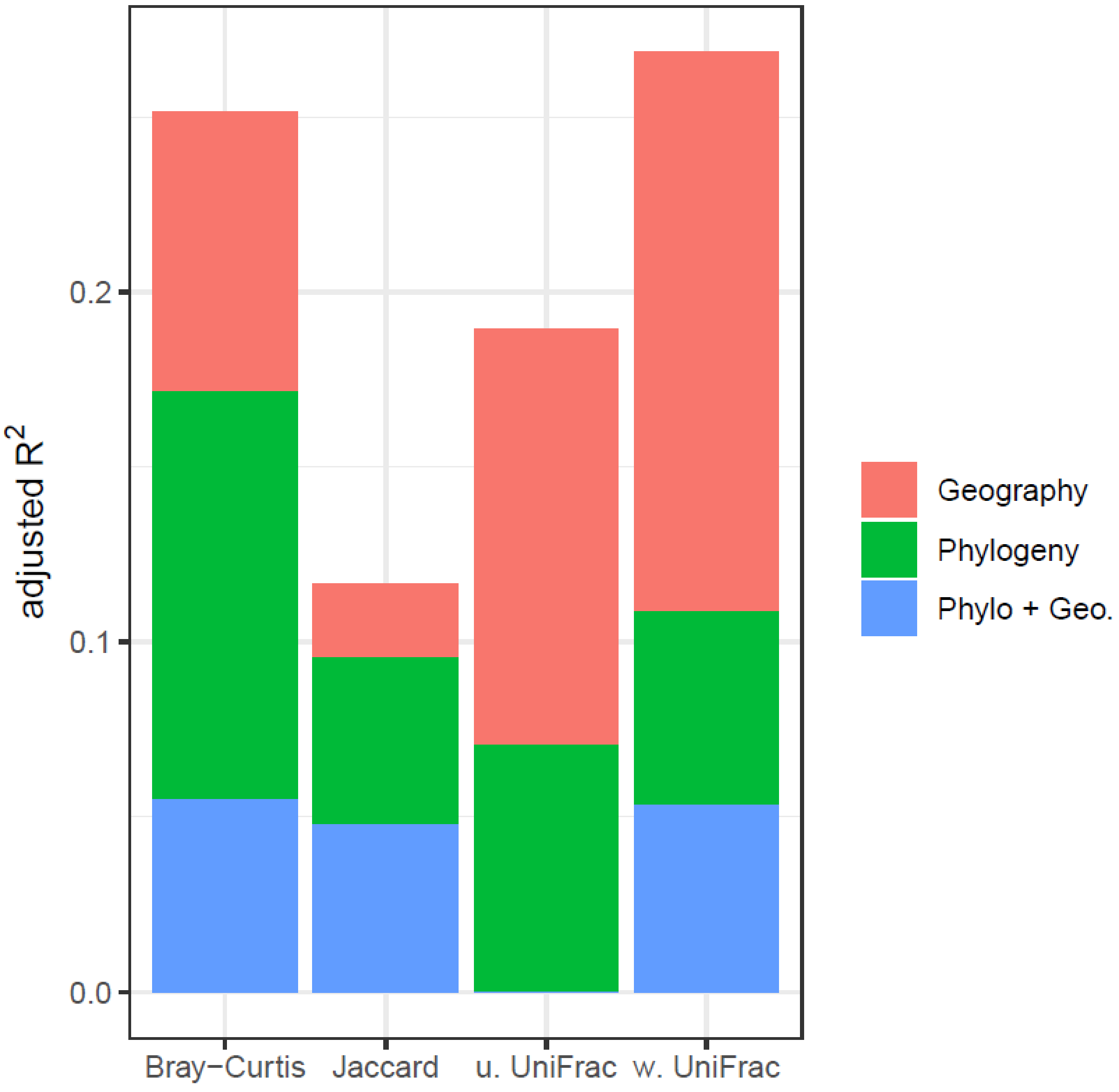

Supplement: Supplementary file 8 — Figure S3 [file 41396_2019_438_MOESM8_ESM.pdf]
